# Atomic resolution view into the structure–function relationships of the human myelin peripheral membrane protein P2

**DOI:** 10.1107/S1399004713027910

**Published:** 2013-12-31

**Authors:** Salla Ruskamo, Ravi P. Yadav, Satyan Sharma, Mari Lehtimäki, Saara Laulumaa, Shweta Aggarwal, Mikael Simons, Jochen Bürck, Anne S. Ulrich, André H. Juffer, Inari Kursula, Petri Kursula

**Affiliations:** a Department of Biochemistry, University of Oulu, Oulu, Finland; b Biocenter Oulu, University of Oulu, Oulu, Finland; c Molecular Biology Unit, Institute of Medical Sciences (IMS), Banaras Hindu University, Varanasi, India; d Centre for Structural Systems Biology, Helmholtz Centre for Infection Research (CSSB-HZI), German Electron Synchrotron (DESY), Hamburg, Germany; e Max Planck Institute for Experimental Medicine, Göttingen, Germany; f Institute of Biological Interfaces (IBG-2), Karlsruhe Institute for Technology (KIT), Karlsruhe, Germany; g Department of Chemistry, University of Hamburg, Hamburg, Germany

**Keywords:** human myelin peripheral membrane protein P2, myelin, membrane proteins

## Abstract

P2 is a fatty acid-binding protein expressed in vertebrate peripheral nerve myelin, where it may function in bilayer stacking and lipid transport. P2 binds to phospholipid membranes through its positively charged surface and a hydrophobic tip, and accommodates fatty acids inside its barrel structure. The structure of human P2 refined at the ultrahigh resolution of 0.93 Å allows detailed structural analyses, including the full organization of an internal hydrogen-bonding network. The orientation of the bound fatty-acid carboxyl group is linked to the protonation states of two coordinating arginine residues. An anion-binding site in the portal region is suggested to be relevant for membrane interactions and conformational changes. When bound to membrane multilayers, P2 has a preferred orientation and is stabilized, and the repeat distance indicates a single layer of P2 between membranes. Simulations show the formation of a double bilayer in the presence of P2, and in cultured cells wild-type P2 induces membrane-domain formation. Here, the most accurate structural and functional view to date on P2, a major component of peripheral nerve myelin, is presented, showing how it can interact with two membranes simultaneously while going through conformational changes at its portal region enabling ligand transfer.

## Abstract 2

The structure of the human myelin peripheral membrane protein P2 has been refined at 0.93 Å resolution. In combination with functional experiments *in vitro*, *in vivo* and *in silico*, the fine details of the structure–function relationships in P2 are emerging.

## 1. Introduction   

The myelin sheath is a unique domain of a glial cell plasma membrane which forms tightly packed, ordered and multilayered proteolipid structures that wrap around selected axons in the nervous system. Myelin contains very little solvent and carries a set of specific proteins that are present at high concentration and are thought to mediate myelin membrane stacking and stabilization. Most myelin proteins are involved in neurological diseases, but current knowledge about their structure–function relationships is limited (Kursula, 2008 ▶; Han *et al.*, 2013 ▶).

Myelin protein 2 (P2) is a small highly basic protein of the fatty acid-binding protein (FABP) family that is expressed in peripheral nerve myelin sheaths in high abundance (Trapp *et al.*, 1984 ▶). The FABP family includes proteins involved in fatty-acid transport (Storch & Thumser, 2000 ▶). For most FABPs, a collisional transfer model of ligands to/from the membrane has either been suggested or detected (Storch & Thumser, 2000 ▶), involving a direct interaction of the protein with the lipid bilayer, as well as electrostatic and hydrophobic forces between the helical lid domain and the membrane (Storch & McDermott, 2009 ▶). The functional details of P2 in this respect have not been studied, although it is assumed to use the collisional mechanism.

P2 is thought to be involved in the stacking of two apposing cytoplasmic membrane leaflets in the compacted myelin membrane (Sedzik *et al.*, 1985 ▶; Suresh *et al.*, 2010 ▶). It may also be involved in the stabilization of peripheral nerve myelin in Shiverer mutant mice, which lack another major myelin protein, the myelin basic protein (MBP; Winter, 1982 ▶). P2 has been suggested to be a potential autoantigen in Guillain–Barré syndrome, an autoimmune peripheral neuropathy (Rostami, 1997 ▶; Hughes *et al.*, 1999 ▶). While P2 is known to bind to lipid membrane surfaces, inducing their stacking (Sedzik *et al.*, 1985 ▶; Suresh *et al.*, 2010 ▶), and to affect lipid bilayer dynamics (Knoll *et al.*, 2010 ▶), its membrane-binding mechanism is unknown at the molecular level.

Our aim was to obtain an atomic resolution view into the interactions of human P2 with both membrane surfaces and monomeric lipids. A combination of ultrahigh-resolution X-­ray crystallography, molecular-dynamics (MD) simulations, cell biology and biophysical analyses yields a picture of P2 structure–function relationships with unprecedented accuracy.

## 2. Experimental procedures   

### 2.1. Protein expression and purification   

The expression and purification of human P2, as well as its mutagenesis, have been described elsewhere (Majava *et al.*, 2010 ▶; Lehtimäki *et al.*, 2012 ▶). Briefly, P2 was expressed in *Escherichia coli* Rosetta(DE3) cells using the autoinduction method (Studier, 2005 ▶) with a culture temperature of 310 K for 4 h and then 291 K for 48 h. The protein was purified using metal-ion affinity and size-exclusion chromatography, and the His tag was removed with TEV protease (van den Berg *et al.*, 2006 ▶). The L27D mutant was purified using an identical protocol. His-tagged mouse 18.5 kDa MBP C1 isoform was expressed and purified as described previously (Bates *et al.*, 2000 ▶; Suresh *et al.*, 2010 ▶).

### 2.2. Crystal structure determination and refinement   

For crystallization, conditions similar to our previous study (Majava *et al.*, 2010 ▶) were used. The optimal crystallization conditions were found using hanging-drop vapour diffusion at 277 K. The well solution consisted of 100 m*M* sodium citrate pH 5.0, 26%(*w*/*v*) polyethylene glycol (PEG) 6000 and the protein was in a buffer consisting of 25 m*M* HEPES pH 7.5, 250 m*M* NaCl, 10% glycerol, 0.5%(*w*/*v*) cholesterol. For crystallization, 1 µl protein solution and 1 µl well solution were mixed and equilibrated against 500 µl well solution. Cholesterol was included in the crystallization buffer, since the possibility of obtaining a crystal of a P2–cholesterol complex was being screened. However, no electron density for cholesterol was detected in the crystal structure, and the same tightly packed crystal form could subsequently be grown without lipidic additives.

The morphology of the crystals was rod-like, visually differing from the previous bipyramidal crystal form. For cryoprotection, 1 µl well solution and 1 µl 100% PEG 200 were added to the crystallization drop prior to picking up the crystal.

X-ray diffraction data were collected on the synchrotron-radiation beamline I911-3 at MAX-lab, Lund, Sweden at 100 K. Data were processed using *XDS* (Kabsch, 2010 ▶) and *XDSi* (Kursula, 2004 ▶). While the space group of the crystal was *P*4_1_2_1_2, as seen previously (Majava *et al.*, 2010 ▶), the *a* and *b* axes in the new crystal form were more than 12% shorter than before.

The structure was solved using the 1.8 Å resolution structure of human P2 (PDB entry 2wut; Majava *et al.*, 2010 ▶) as a model in *Phaser* (McCoy *et al.*, 2007 ▶). Structure refinement was performed in *phenix.refine* (Adams *et al.*, 2010 ▶) and *REFMAC* (Murshudov *et al.*, 2011 ▶), and model building was performed in *Coot* (Emsley *et al.*, 2010 ▶). H atoms were added at riding positions, and all non-H atoms were refined with anisotropic ADPs. The final model was refined to a resolution of 0.93 Å; the data-collection and refinement statistics are given in Table 1 ▶. The data were cut at 0.93 Å resolution, where 〈*I*/σ(*I*)〉 was above 1, and both CC_1/2_ (Karplus & Diederichs, 2012 ▶; Diederichs & Karplus, 2013 ▶) and the refinement *R* factors in the highest resolution shell indicated significant and useful information content even at this resolution and possibly even beyond (Table 1 ▶). The somewhat low completeness and multiplicity in the very highest resolution shell resulted from data collection on a square detector. The atomic coordinates and structure factors were deposited in the PDB (entry 4bvm). The structure was further validated and analyzed using *MolProbity* (Chen *et al.*, 2010 ▶), *DSSP* (Kabsch & Sander, 1983 ▶), *PyMOL*, *PARVATI* (Merritt, 1999 ▶), *UCSF Chimera* (Pettersen *et al.*, 2004 ▶) and *MSMS* (Sanner *et al.*, 1996 ▶).

### 2.3. Surface plasmon resonance   

The binding of P2 to lipid layers was followed by surface plasmon resonance (SPR) using a Biacore T100 instrument (GE Healthcare). Dimyristoyl phosphatidic acid (DMPA) was immobilized on an HPA chip (GE Healthcare) as a monolayer according to the manufacturer’s protocol, and the binding of P2 was assessed by injecting concentrations of between 50 n*M* and 2 µ*M* onto the chip. The running buffer was 10 m*M* HEPES pH 7.4, 150 m*M* NaCl and experiments were carried out at 298 K. As a control, bovine serum albumin (BSA) was used to confirm the absence of nonspecific binding. The response at equilibrium was plotted against P2 concentration to obtain an estimate of binding affinity.

### 2.4. Liquid-state and oriented circular dichroism   

#### 2.4.1. Sample preparation for circular dichroism   

P2 was dialyzed into 10 m*M* phosphate buffer pH 7.0 (PB). Dimyristoyl phosphatidylcholine (DMPC) and dimyristoyl phosphatidylglycerol (DMPG) were used for liposome preparation (Avanti Polar Lipids, Alabaster, Alabama, USA). The lipids were separately dissolved in 1:1(*v*:*v*) chloroform/methanol to obtain individual lipid stock solutions of 7.3 m*M*. Aliquots of the stock solutions were combined and vortexed to obtain a 1:1 molar DMPC/DMPG mixture. Subsequently, organic solvents were removed under a gentle stream of nitrogen, followed by 4–5 h in a vacuum. The thin DMPC/DMPG lipid film was dispersed in PB and the lipid dispersion was homogenized and hydrated by vigorous vortexing for 7 × 1 min and by performing seven freeze–thaw cycles, resulting in multilamellar vesicles (MLVs). Small unilamellar vesicles (SUVs) were generated by sonication of the MLVs for 3 × 4 min in a strong ultrasonic bath (UTR200, Hielscher, Germany), avoiding overheating of the samples.

For preparing the solution CD samples, P2 was diluted with either PB or with the liposome dispersion in PB. The protein concentration in the final samples was 10 µ*M* (0.15 mg ml^−1^), while the lipid concentration was 1.5 m*M*, resulting in a protein:lipid molar ratio of 1:150.

For oriented CD (OCD) in 1:1 DMPC/DMPG bilayers, 200 µl of the P2/lipid SUVs were deposited as a spot of ∼12 mm diameter on a UV-transparent quartz glass plate (Suprasil QS, Hellma Optik GmbH, Jena, Germany) and water was evaporated under a gentle stream of air. The amount of deposited lipid was 0.3 µmol and the total amount of P2 was 1.95 nmol. The quartz window with the dried sample was assembled with a second clean window in the OCD sample cell. The glass-enclosed, air-exposed lipid film was rehydrated for 15 h at 303 K and 97% relative humidity using saturated K_2_SO_4_. During rehydration, the lipids spontaneously align as macroscopically oriented lipid bilayers on the glass surface.

#### 2.4.2. Conventional and oriented CD spectropolarimetry   

CD spectra of P2 in PB and 1:1 DMPC/DMPG SUVs were recorded on a J-815 spectropolarimeter (Jasco, Gross-Umstadt, Germany). Measurements were performed in a 1 mm quartz cell (Suprasil, Hellma) between 260 and 190 nm at 0.1 nm intervals. Spectra were recorded at 293 K without and at 303 K with the SUVs, *i.e.* above the phase-transition temperature of the lipids. Three scans at a scan rate of 10 nm min^−1^ with 8 s response time and 1 nm bandwidth were averaged for each sample and baseline, and the baseline was subtracted from the sample spectrum. Mean residue ellip­ticities (MRE) were then calculated based on the P2 concentration.

For oriented CD analysis, a computer-controlled OCD cell (Bürck *et al.*, 2008 ▶) was used that can be integrated into a J-810 spectropolarimeter as an accessory, allowing measurements under controlled humidity (97%) and temperature (303 K). The optical path runs along the cylindrical axis of the cell, *i.e.* normal to the quartz glass window surface carrying the oriented lipid film. OCD spectra were recorded in steps of 45° by rotating the sample cell about the beam axis (Chen *et al.*, 2002 ▶) to reduce possible spectral artifacts caused by linear dichroism or linear birefringence owing to imperfections in the sample, strain in the quartz glass windows or imperfect alignment of the windows (Olah & Huang, 1988 ▶; Wu *et al.*, 1990 ▶). At each angle, three scans were recorded and averaged using the same data-acquisition parameters as above. The eight rotational spectra were subsequently averaged and the background spectra of lipid bilayers without P2 were subtracted. For determining the MRE values of the P2 OCD spectrum, the absorption maximum around 192 nm of P2 in 1:1 DMPC/DMPG vesicles and in the oriented bilayers was used to calculate a correction factor owing to the fact that the exact thickness of the stacked lipid bilayers and the ‘molar concentration’ of P2 in the lipid bilayer film was unknown.

#### 2.4.3. Thermal stability analysis   

The thermal stability of human P2 in the presence and absence of DMPC/DMPG vesicles was studied using a Chirascan Plus spectropolarimeter (Applied Photophysics). The protein concentration was 0.25 mg ml^−1^ and the lipids were in a 100-fold molar excess. The quartz cell had a path length of 0.5 mm and the buffer consisted of 0.8 m*M* HEPES pH 7.5, 6 m*M* NaCl, 0.4% glycerol. Spectra were measured continuously during heating from 293 to 363 K at a heating rate of 1 K min^−1^ and experiments were carried out with identical settings in the presence and absence of the vesicles.

### 2.5. Coarse-grained simulations with lipids   

The atomistic structure of human P2 (Majava *et al.*, 2010 ▶) was converted to a coarse-grained (CG) representation using the standard MARTINI force field (Marrink *et al.*, 2007 ▶; Monticelli *et al.*, 2008 ▶). The CG protein was placed in a random orientation above a well equilibrated CG bilayer composed of 127 molecules of both dipalmitoyl phospha­tidylcholine (DPPC) and dipalmitoyl phosphatidylglycerol (DPPG). The centre of mass of the protein was ∼50 Å away from the centre of mass of the bilayer. The box was increased in the *Z* dimension and a further 127 DPPC and 127 DPPG molecules were added randomly in successive steps. All manipulations were performed with *VMD* (Humphrey *et al.*, 1996 ▶). The system was then solvated in water. Water molecules in the interior of the pre-formed bilayer were removed and appropriate counterions were added to preserve charge neutrality of the system.

Molecular-dynamics simulations were performed with *GROMACS* 4 (Hess *et al.*, 2008 ▶). The simulations were run under NpT conditions (323 K, 1 atm) using the Berendsen algorithm (Berendsen *et al.*, 1984 ▶) with a coupling constant of 0.5 ps for temperature and 5 ps for pressure. The lipids in the two bilayers, the protein and the water were separately coupled to thermostats. The whole system was coupled to the barostat semi-isotropically (across *XY*/*Z*). Lennard–Jones interactions were cut off at 12 Å and long-range electrostatics were handled using the particle mesh Ewald method (Essmann *et al.*, 1995 ▶). A time step of 25 fs was used for integrating the equations of motion.

### 2.6. Atomistic simulations   

The crystal structure of the palmitate–P2 complex (PDB entry 2wut; Majava *et al.*, 2010 ▶) was used as the starting structure for atomistic simulations with and without bound ligand. The fatty-acid coordinates were removed for the apo P2 simulations. All simulations were performed using *GROMACS* 4.0.7 (Hess *et al.*, 2008 ▶) with the ffG53a6 force field (Oostenbrink *et al.*, 2004 ▶). The topology of palmitate was based on the DPPC topology described using Berger parameters (Berger *et al.*, 1997 ▶). Each system was then solvated in a bath of simple point charge water molecules (Berendsen *et al.*, 1981 ▶) and subjected to steepest-descent energy minimization until a convergence value of 100 kJ mol^−1^ nm^−1^ was reached. The system was subjected to a production MD run using an integration step of 2 fs. The linear constraint solver (LINCS) method was used to constrain the bond lengths (Hess *et al.*, 1997 ▶). Electrostatic interactions were calculated using the particle mesh Ewald method (Darden *et al.*, 1993 ▶; Bussi *et al.*, 2007 ▶) with a 1.0 nm cutoff. NpT conditions (constant number of particles, pressure and temperature) were used in the simulations. The temperature (300 K) and pressure (1 atm) were controlled using the V-rescale algorithm (Bussi *et al.*, 2007 ▶) and the Parrinello–Rahman barostat (Parrinello & Rahman, 1981 ▶).

### 2.7. Vesicle aggregation and small-angle X-ray diffraction   

Aggregation of lipid vesicles was followed by turbidimetry, comparing the wild type and the L27D mutant of P2. Different amounts of P2 were mixed with 0.5 m*M* DMPC/DMPG vesicles and the turbidity was measured in 10 m*M* HEPES pH 7.4, 150 m*M* NaCl by recording the absorbance at 600 nm using a Tecan Infinite M200 plate reader.

For measuring the bilayer repeat distance in membranes stacked by P2 and MBP, small-angle X-ray scattering data were collected from solution samples of aggregated vesicles using synchrotron radiation on the I911-4 beamline at MAX-­lab (Lund, Sweden). The concentrations used for this experiment were 2 m*M* lipid (1:1 DMPC/DMPG) and 20 µ*M* P2 (molar ratio 1:100). For MBP, 2 µ*M* protein was mixed with 1–3 m*M* lipid (molar ratio 1:500–1:1500). For preparing the samples, both MBP and P2 were dialyzed against water and were then mixed with the DMPC/DMPG vesicle preparation. The milky appearance upon mixing gave initial evidence for vesicle aggregation. In the diffraction experiment, the vesicle preparations without protein did not give rise to any Bragg peaks, whereas distinct peaks were observed for samples containing P2 or MBP. The peak positions could further be used to infer the repeat distance in the stacked membranes using the relationship *d* = 2π/*s*, where *d* is the repeat distance and *s* is the momentum transfer, which in turn is defined as *s* = 4πsinθ/λ, where θ is the scattering angle and λ is the X-ray wavelength.

### 2.8. Functional assay in cell culture   

The membrane interactions induced by wild-type and L27D mutant P2 in a cellular model system were studied by overexpressing green fluorescent protein (GFP)–transmembrane domain (TM)–P2 fusion proteins in Ptk2 cells, followed by fluorescence microscopy, essentially as described by Aggarwal *et al.* (2013 ▶). The detection of fluorescent membrane domains in this assay is a sign of specific contacts being formed between the endoplasmic reticulum and the plasma membrane.

## 3. Results and discussion   

### 3.1. The atomic resolution structure of human P2   

The crystal structure of human P2 was refined at an ultrahigh resolution of 0.93 Å (Table 1 ▶). This is by far the highest resolution achieved for any FABP. The crystal form is related to that previously used to determine the structure at 1.8 Å resolution (Majava *et al.*, 2010 ▶), but the new crystals display much tighter packing, higher ordering and better diffraction.

**Figure 1:**
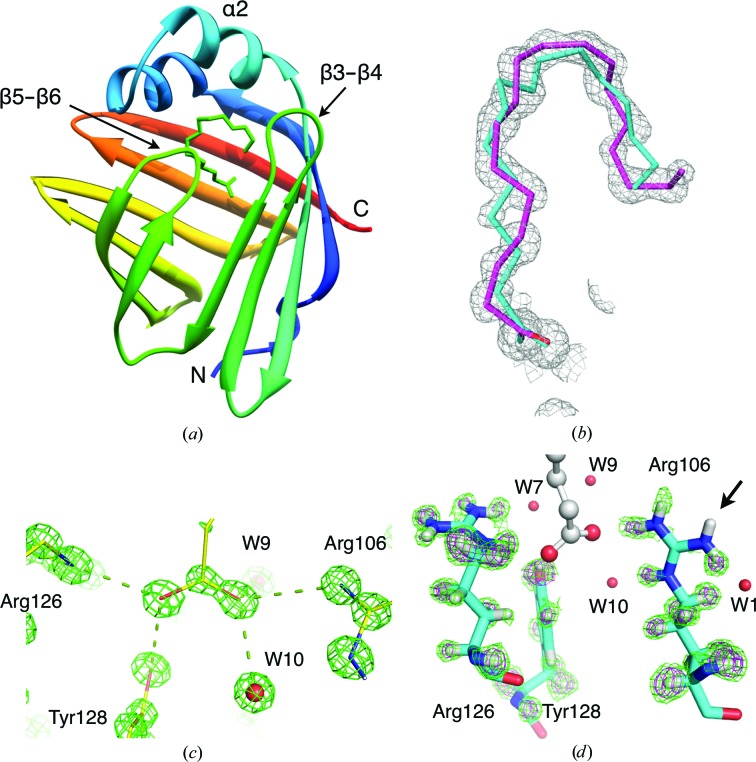
Crystal structure of human P2. (*a*) Overall structure of human P2. (*b*) Electron density for the ligands *cis*-vaccenate (magenta) and palmitate (cyan). The final refined 2*F*_o_ − *F*_c_ map is contoured at 1.5σ. OMIT maps calculated without palmitate are shown in Supplementary Fig. S2. (*c*) Orientation of the fatty-acid COOH group. The 2*F*_o_ − *F*_c_ map (5.5σ) is shown. (*d*) The difference map, calculated without H atoms (green, 1.7σ; magenta, 2.5σ). The lost proton in Arg106 is indicated by the arrow.

The overall fold of human P2, comprising a twisted ten-stranded β-barrel and an α-helical lid, is similar to other conserved FABPs (Fig. 1 ▶ *a*). The ultrahigh-resolution crystallographic data now enable a detailed analysis of the human P2 structure and its ligand-binding determinants. The 16-carbon palmitate, which is very abundant in *E. coli*, is in fact not the only fatty acid present in the crystal, as was concluded earlier based on the 1.8 Å resolution electron density (Majava *et al.*, 2010 ▶); there is an additional partial occupancy of an 18-carbon fatty acid (Fig. 1 ▶ *b*, Supplementary Fig. S2^1^). The additional electron density corresponds well to the presence of *cis*-vaccenate, another highly abundant fatty acid in *E. coli* (Gelmann & Cronan, 1972 ▶). Hence, recombinant P2 expressed in *E. coli* can form stable complexes with both saturated and non-saturated fatty acids from the expression host.

The high resolution reveals accurate details of the liganded P2 complex, including the positions of a number of H atoms involved in ligand interactions and in the ordered water network inside the binding cavity. The orientation of the fatty-acid carboxyl group can be unambiguously defined based on the electron density (Fig. 1 ▶ *c*). The double-bonded O atom interacts with Arg106 and water molecules 9 and 10, while the single-bonded O atom contacts Arg126 and Tyr128. Interestingly, careful observation of the difference electron-density maps reveals that the two arginine residues in the binding cavity have different protonation states: Arg126 is protonated as expected, while Arg106 is clearly in the deprotonated neutral form (Fig. 1 ▶ *d*). This coupling of the fatty-acid orientation and arginine protonation state could be a common feature of FABPs.

**Figure 2:**
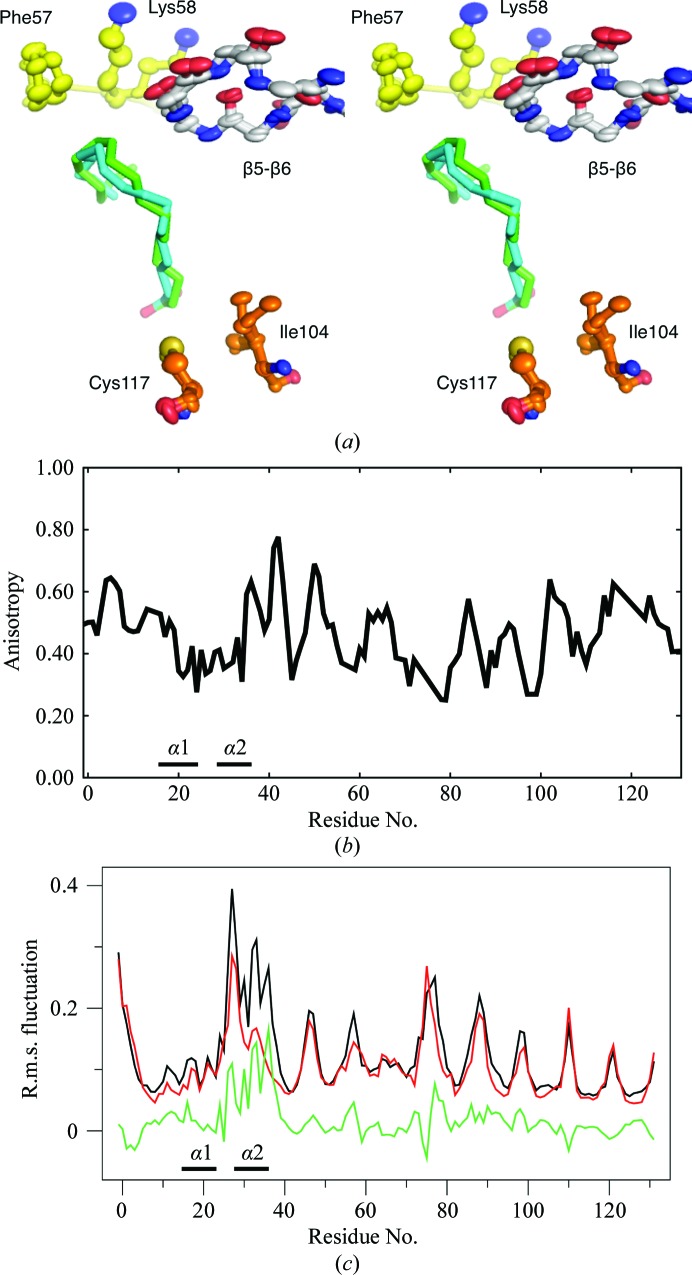
Anisotropy and dynamics in human P2. (*a*) Alternative conformations in residues interacting with the fatty acid in the binding cavity. (*b*) Main-chain mean anisotropy along the human P2 sequence. A fully isotropic atom has an anisotropy of 1.0. (*c*) Atomistic simulation of apo (black) and liganded (red) P2 based on a 30 ns trajectory. The difference between the apo and liganded simulations is shown in green.

Several alternative conformations, also in the area of the ligand-binding pocket, are evident (Fig. 2 ▶ *a*). This flexibility is likely to be related to the identity of the bound ligand. In addition, high anisotropy can be observed even in the main chain for parts of the portal region, for example in the loops β5–β6 and β7–β8 as well as in the helical lid (Figs. 2 ▶ *a* and 2 ▶ *b*). The data indicate flexibility in regions of P2 interacting with the bound ligands, highlighting the possibility of adopting a variety of hydrophobic ligands inside P2.

The effect of a bound fatty acid on P2 dynamics was studied by atomistic MD simulations (Fig. 2 ▶ *c*), which show that helix α2 is more dynamic in the absence of fatty acid. The presence of a bound palmitate molecule significantly stabilizes the helical lid, especially its tip and helix α2. This is analogous to the stabilization of helix α2 in FABP1 upon lipid binding observed using NMR spectroscopy (Cai *et al.*, 2012 ▶). In addition to the lid, the loops in the portal region are among the most dynamic in P2, although the presence of the ligand has only minor effects on them. The simulations hence imply inherent flexibility in the regions assumed to take part in cavity opening and membrane binding.

### 3.2. Internal water structure   

**Figure 3:**
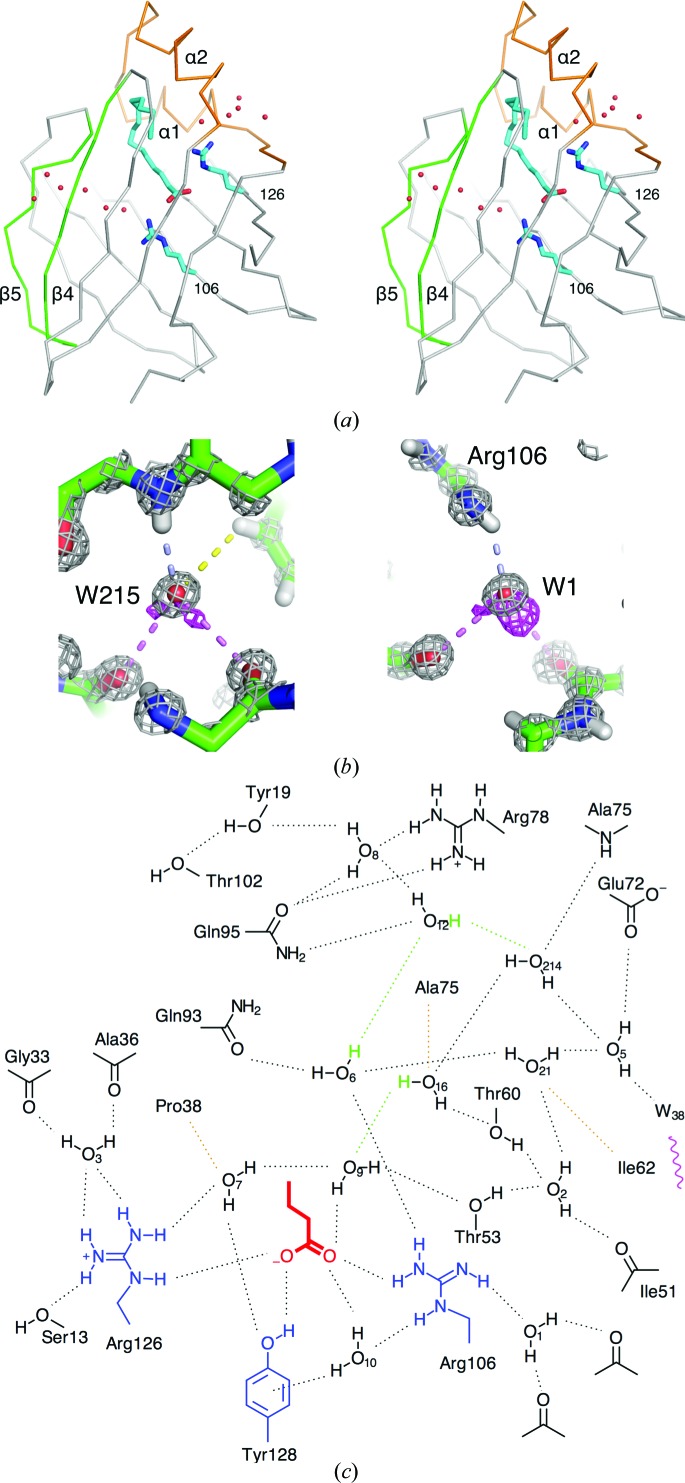
Organization of internal water molecules. (*a*) Two water channels connecting the ligand-binding cavity to bulk solvent. (*b*) Two structural water molecules in human P2. Left: the orientation of water 215. Grey, 2*F*_o_ − *F*_c_ map at 4.0σ; magenta, *F*_o_ − *F*_c_ map at 2.4σ. Right: water 1. Grey, 2*F*_o_ − *F*_c_ map at 4.0σ; magenta, *F*_o_ − *F*_c_ map at 2.7σ. The hydrogen bonds donated by the water are shown in pink and those accepted are in light blue. The potential C—H⋯O bond between water 215 and Phe70 is indicated in yellow. (*c*) The hydrogen-bonding network involving the internal water molecules. Only three H atoms from water molecules were not seen in electron density and their respective positions can be deduced geometrically (green). The observed C—H⋯O bonds are shown in orange. The three central residues for fatty-acid binding are in blue and the polar terminus of the fatty acid is in red. Water 38 is connected to bulk solvent (magenta wave) through the channel between β-sheets 4 and 5.

The β-barrel of P2 is not continuous; rather, polar contacts between strands β4 and β5 are mediated by water molecules, which form a solvent channel through the wall of the barrel (Fig. 3 ▶ *a*). Strands β4 and β5 do not in fact have a single main-chain hydrogen bond between them. In the atomic resolution structure strand β5 is mobile; *e.g.* the central Thr74 has a double conformation involving the peptide backbone. The water channel can in principle provide a connection between bulk solvent and the interior water cluster, even when the helical lid is closed and a fatty acid is bound. The discontinuity of the barrel may also play a role in protein flexibility upon ligand exchange in P2 and other FABPs.

Another water channel is formed between the two helices of the portal region, possibly providing the flexibility required for conformational changes of the helical lid. Both of the fatty acid-coordinating Arg residues are directly connected to these water channels (Fig. 3 ▶ *a*). The exact role of the two water channels in P2 function is currently unknown, but they could provide routes for movement of water molecules upon lipid binding.

A structural water molecule was reported in the FABP family, but its presence in P2 was not studied (Likić *et al.*, 2000 ▶). This water molecule (water 215) is also present in P2; it donates hydrogen bonds to the backbone carbonyl atoms of Lys65 and Gln68, and it accepts a hydrogen bond from the backbone amide of Val84, as well as a potential C—H⋯O hydrogen bond from Phe70 (Fig. 3 ▶ *b*). Another water molecule with a putative structural role, water 1, is buried under the side chain of Arg106. Additionally, water 1 makes hydrogen bonds to the backbone carbonyl groups of Leu91 and Ile104 (Fig. 3 ▶ *b*). A corresponding water molecule is present in most other FABPs in which Arg106 is conserved, and is likely to play a role in orienting this deprotonated ligand-binding residue.

By combining the information from different electron-density maps, a complete picture of the hydrogen-bonding network inside the P2 barrel can be obtained (Fig. 3 ▶ *c*). All of the water molecules in the network both donate and accept two hydrogen bonds. It is noteworthy that three of the water molecules act as acceptors of weak C—H⋯O hydrogen bonds from hydrophobic side chains. No such bonds are observed between the bound fatty acid and water in the binding cavity.

### 3.3. Structural implications for membrane binding   

**Figure 4:**
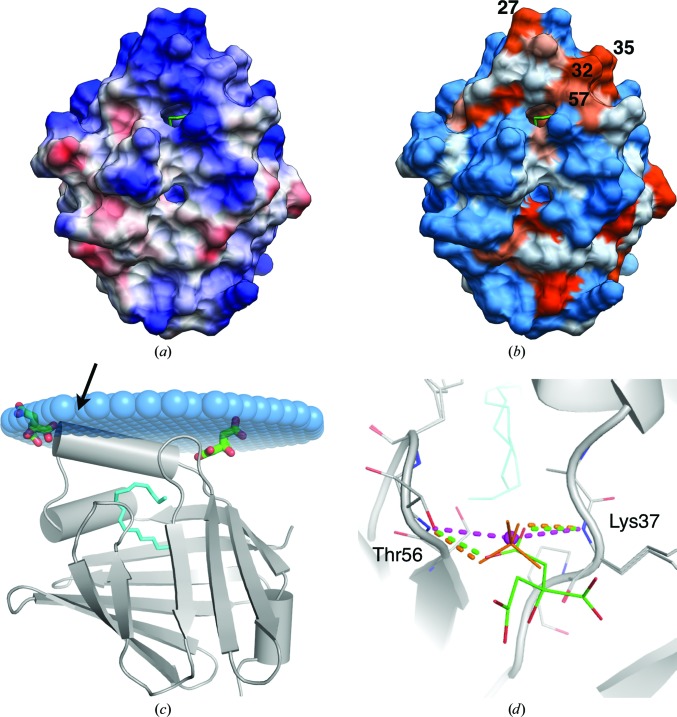
Surface properties of human P2. (*a*) The P2 surface coloured according to electrostatic potential. (*b*) Surface colouring according to the Kyte–Doolittle scale, where orange denotes hydrophobic residues and blue polar residues. The protein is in the same orientation in both images and in Fig. 1 ▶(*a*), with the portal region at the top. (*c*) Predicted membrane-binding mode of human P2 (Lomize *et al.*, 2012 ▶). The two bound citrate molecules are indicated in green. The position of Leu27 at the tip of the helical lid is indicated by an arrow. (*d*) Comparison of the anion-binding site in structures of human P2. Our earlier structure (Majava *et al.*, 2010 ▶) contains a bound chloride ion (magenta) and another recent structure of human P2 (PDB entry 3nr3) contains a sulfate ion (orange). The new structure has citrate (green) bound at the same site.

For binding to a phospholipid bilayer, a peripheral membrane protein is expected to carry patches of positively charged and hydrophobic residues on its surface. While the positive charges interact directly with lipid headgroup phosphate moieties, large hydrophobic side chains could insert deeper into the membrane. P2 exhibits two positively charged patches on opposite faces of the molecule, which could be involved in the stacking of two membranes (Fig. 4 ▶ *a*). The helical lid also contains hydrophobic residues pointing outwards, poised for insertion into the membrane (Fig. 4 ▶ *b*). Prediction of the binding mode to a single membrane surface (Lomize *et al.*, 2012 ▶) suggests partial penetration of the helical lid into the membrane (Fig. 4 ▶ *c*). The β3–β4 loop, including Phe57 and Lys58, is also close to the membrane surface.

It is likely that the main membrane-binding site of P2 is comprised of the helical lid, which has a hydrophobic tip, with Leu27, Leu32 and Leu35 pointing out and a positively charged rim. Membrane binding could lead to lipid transfer to/from the cavity, similarly to the suggested collisional transfer in other FABPs (Thumser *et al.*, 2001 ▶). Lipid transfer would require a conformational change in the portal region, which is also supported by our CD data (see below). On the opposite face of P2 no opening of the barrel is envisaged, and the P2–membrane interaction is most likely to be based on electrostatics.

Anion-binding sites in crystal structures may correspond to sites for interaction with membrane phospholipids. Two citrate molecules are bound to the surface of P2 (Fig. 4 ▶ *c*), close to the hinge and tip areas of the α-­helical lid. That in the hinge region is substituted by either a chloride or a sulfate ion in other human P2 crystal structures (Fig. 4 ▶ *d*). This suggests that small ligands in close proximity to the portal region may mimic the headgroups of phospholipids in biological membranes.

**Figure 5:**
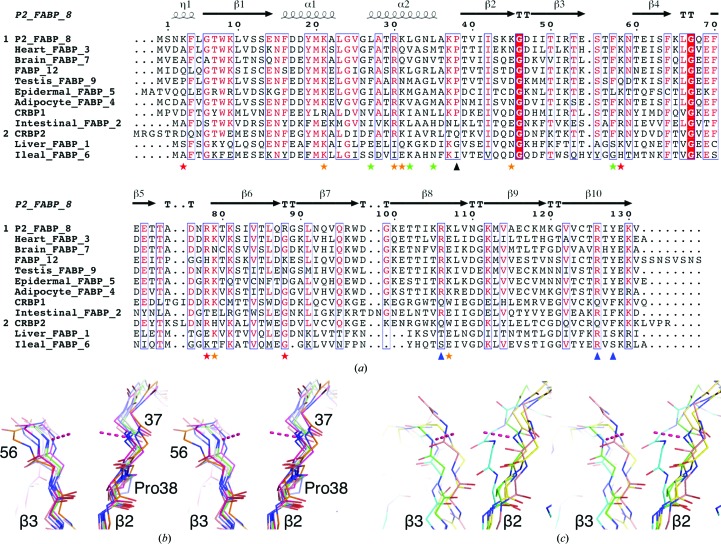
Conserved features in FABP sequences and structures. (*a*) Sequence alignment of the human FABP family. Secondary-structure elements of P2 are shown at the top and the following features are highlighted at the bottom: Pro38 (black triangle), fatty acid-coordinating polar residues (blue triangles), main hydrophobic membrane contacts (green asterisks), main hydrophilic contacts (red asterisks) and other hydrophilic contacts (orange asterisks). See Fig. 8 ▶(*b*) and Supplementary Fig. S1 for details of the contacts obtained using MD. (*b*) The geometry at the anion-binding site in FABPs with a collisional mechanism and a conserved Pro38. (*c*) The same site in FABPs without Pro38. The dashed lines indicate coordination of the bound citrate in the P2 structure. The following PDB entries were superimposed on the human P2 structure: 3vg7 (FABP1; Sharma *et al.*, 2012 ▶), 1ifc (FABP2; Scapin *et al.*, 1992 ▶), 1hmr (FABP3; Young *et al.*, 1994 ▶), 3p6d (FABP4; J. M. Gonzalez & E. Pozharski, unpublished work), 1b56 (FABP5; Hohoff *et al.*, 1999 ▶), 3elx (FABP6; Capaldi *et al.*, 2009 ▶), 1fdq (FABP7; Balendiran *et al.*, 2000 ▶), 4a60 (FABP9; J. R. C. Muniz, W. Kiyani, L. Shrestha, D. S. Froese, T. Krojer, M. Vollmar, C. H. Arrowsmith, A. M. Edwards, J. Weigelt, C. Bountra, F. Von Delft & W. W. Yue, unpublished work), 1crb (CRBP1; Cowan *et al.*, 1993 ▶) and 2rct (CRBP2; Tarter *et al.*, 2008 ▶).

The crystallographic anion-binding site is formed by the backbone amide groups of Lys37 and Thr56, which point towards each other (Fig. 4 ▶ *d*). Upon interaction with the membrane surface, the binding of an anionic group to the portal region could induce conformational changes required for fatty-acid transfer. It is possible that a vicinal proline residue, Pro38, plays a role in keeping these backbone amides facing each other and in regulating lid opening. Considering the possible lipid-transfer modes in the FABP family – diffusive and collisional – the best-characterized FABP with a diffusive mechanism is FABP1 (liver FABP; Thumser & Storch, 2000 ▶). In FABPs with a reported or presumed collisional mechanism and a known three-dimensional structure, Pro38 is conserved (Fig. 5 ▶ *a*), and the backbone amides of residues 37 and 56 are oriented as in P2 (Fig. 5 ▶ *b*). Interestingly, in FABP1 Pro38 is not conserved and the backbone amides forming the anion-binding site in P2 do not face each other (Sharma *et al.*, 2012 ▶; Fig. 5 ▶ *c*). These observations also further apply to the two cellular retinol-binding proteins (CRBPs) CRBP1 and CRBP2, of which CRBP2 has a diffusive mechanism (Herr *et al.*, 1999 ▶); in CRBP2, the backbone conformation is different from P2. The latter is also true for FABP2 and FABP6, in which Pro38 is not conserved (Fig. 5 ▶ *c*).

### 3.4. Binding to lipid membrane surfaces   

**Figure 6:**
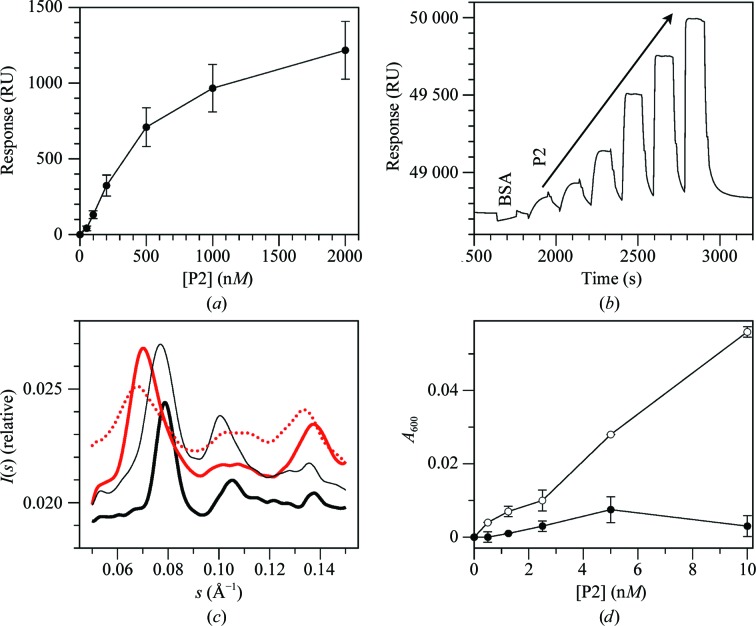
Membrane binding by P2. (*a*) Binding of P2 to DMPA monolayers in SPR. (*b*) A raw SPR sensorgram with injections in rapid succession: 1.5 µ*M* BSA followed by 50, 100, 200, 500, 1000 and 2000 n*M* P2. (*c*) Small-angle X-ray diffraction of P2 and MBP mixed with DMPC/DMPG vesicles: wild-type P2 (solid red line), L27D P2 (dashed red line), MBP at a 1:500 ratio (thick black line) and MBP at a 1:1500 ratio (thin black line). (*d*) Vesicle-aggregation assay of wild-type (open symbols) and L27D (filled symbols) P2.

The binding of P2 to immobilized membrane surfaces was analyzed using SPR. A *K*_d_ value of 0.7 µ*M* for P2 binding to an anionic DMPA monolayer could be estimated (Fig. 6 ▶ *a*). Another myelin peripheral membrane protein, MBP, has been studied in detail with regard to membrane binding and associated conformational changes (Keniry & Smith, 1979 ▶; Ahmed *et al.*, 2010 ▶; Polverini *et al.*, 2011 ▶; Wang *et al.*, 2011 ▶), but is structurally unrelated to P2. P2 was readily dissociated from the surface by simple washing (Fig. 6 ▶ *b*), unlike MBP (Wang *et al.*, 2011 ▶). The apparent affinity of P2 towards membrane surfaces is slightly lower than that of MBP (Wang *et al.*, 2011 ▶), and the binding kinetics of the two proteins differ markedly. While P2 binds rapidly, it also has a very fast off-rate from membranes in SPR compared with MBP (Wang *et al.*, 2011 ▶). This may reflect a deeper penetration of MBP into the membrane than P2, as well as large-scale conformational changes accompanying MBP membrane binding (Wang *et al.*, 2011 ▶).

We used small-angle X-ray diffraction to measure the repeat distances in membrane multilayers induced by P2 and MBP. When mixed with lipid vesicles, both proteins cause aggregation of the vesicles, indicating an ability to stack lipid bilayers together. Ordered stacking is proven by the appearance of Bragg peaks upon X-ray exposure (Fig. 6 ▶ *c*). The calculated repeat distances are 89 Å for P2 and 80–82 Å for MBP. Hence, the lipid membranes are packed more tightly with MBP, but the packing induced by P2 also results in a close apposition of the membranes, with only just enough space for a single layer of P2 in between. The diameter of P2 is 45 Å and the steric bilayer thickness for DMPC is around 44 Å (Balgavý *et al.*, 2001 ▶). Our samples were fully hydrated, and it is likely that somewhat shorter distances apply in a partially hydrated environment.

Based on the crystal structure, the point mutant L27D was prepared (Lehtimäki *et al.*, 2012 ▶) in order to shed light on the importance of the hydrophobic tip of the helical lid in membrane binding. Notably, the Bragg peak was much weaker and broader for the L27D mutant and the repeat distance was 3–4 Å larger than for wild-type P2, suggesting that the mutant induces less stacking with a larger intermembrane space and more disorder in the stacked layers. Turbidimetric analysis of vesicle aggregation also indicated a nearly complete lack of aggregation by the L27D mutant (Fig. 6 ▶ *d*). These results highlight a crucial role of the hydrophobic tip of the P2 helical lid domain in membrane stacking.

### 3.5. Conformation and orientation of P2 in lipid bilayers   

**Figure 7:**
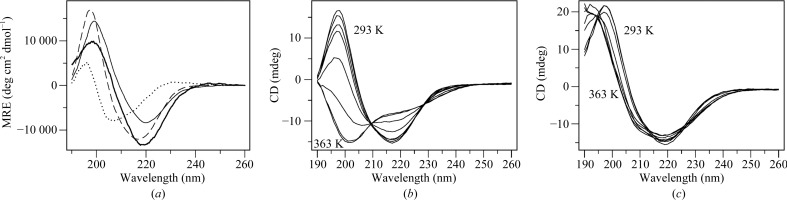
CD analysis of P2. (*a*) CD spectra in buffer (dashed line) and in DMPC/DMPG liposomes (1:150 protein:lipid, thin line). The difference spectrum between these samples is shown as a dotted line and the OCD spectrum of P2 in oriented membranes is shown as a thick line. (*b*, *c*) Thermal stability of P2 in solution (*b*) and of membrane-bound P2 (*c*). For clarity, CD spectra are shown at intervals of 10 K from 293 to 363 K.

Liquid-state CD spectroscopy of human P2 revealed conformational changes in P2 upon membrane binding (Fig. 7 ▶ *a*). In agreement with earlier SRCD measurements (Majava *et al.*, 2010 ▶), the spectrum of P2 in aqueous buffer indicates a predominantly β-­sheet conformation. For comparison, P2 bound to DMPC/DMPG SUVs showed a distinctly different CD lineshape, with the ratio between the maximum and the minimum intensity increasing from 1.4 to 1.7. A difference spectrum suggests the loss of α-helical structure upon membrane binding (Fig. 7 ▶ *a*). As the only α-­helical segment in P2 comprises the helical lid, the data indicate at least partial unfolding of these helices upon membrane binding.

OCD is a method to characterize the orientation of secondary-structure elements in membrane-bound molecules and can, for example, reveal the orientation of α-helical peptides with respect to the membrane (Olah & Huang, 1988 ▶; Wu *et al.*, 1990 ▶; Bürck *et al.*, 2008 ▶). While OCD has been used for studying α-helical membrane-active peptides (Muhle-Goll *et al.*, 2012 ▶; Paulmann *et al.*, 2012 ▶; Steinbrecher *et al.*, 2012 ▶) and helical proteins (Lange *et al.*, 2007 ▶; Nolandt *et al.*, 2009 ▶), limited literature exists on using OCD for β-structures. Only two antimicrobial β-sheet peptides, protegrin (Heller *et al.*, 1998 ▶) and (KIGAKI)_3_ (Wadhwani *et al.*, 2012 ▶), have been examined using OCD in oriented lipid bilayers.

Attempts have been made to characterize the directional dependence of the CD of β-sheet structures in lipid bilayers, for example for poly(Leu-Lys) in phosphatidylcholine (Bazzi *et al.*, 1987 ▶). However, owing to the β-sheet CD tensor being complex (Woody, 1993 ▶) compared with that for an α-helix, and because of the structural variations of β-structures (*e.g.* parallel, antiparallel, twisted, β-helix), no quantitative theory or evaluation procedure yet exist. Therefore, we applied OCD to P2 in oriented bilayers as a qualitative measure. As the OCD spectrum is different from that of the isotropic sample (Fig. 7 ▶ *a*), it can be stated that P2 binds to membranes in a preferred orientation. The full-length protein OCD experiment described here for a β-­barrel structure is the first example of its kind and is a useful starting point for future studies of both P2 binding to membranes and the general use of OCD for β-structured proteins.

CD spectroscopy was further used to assess the stability of human P2 in the presence and absence of vesicles. P2 becomes essentially fully unfolded upon thermal denaturation in aqueous buffer, with an estimated *T*_m_ of 333 K (Fig. 7 ▶ *b*). When bound to lipid vesicles, however, no unfolding is apparent apart from a small conformational change (Fig. 7 ▶ *c*). Thus, a significant stabilization of the folded P2 structure is observed upon membrane binding. Our earlier work indicated that P2 rigidifies the lipid bilayer (Knoll *et al.*, 2010 ▶). Hence, bilayer stacking by P2 results in the stabilization of both the protein and lipid phases of the multilayered membrane. This may be an important functional aspect of P2, in view of the very long lifespan of a mature myelin sheath.

### 3.6. Membrane interaction of human P2 *in silico*   

**Figure 8:**
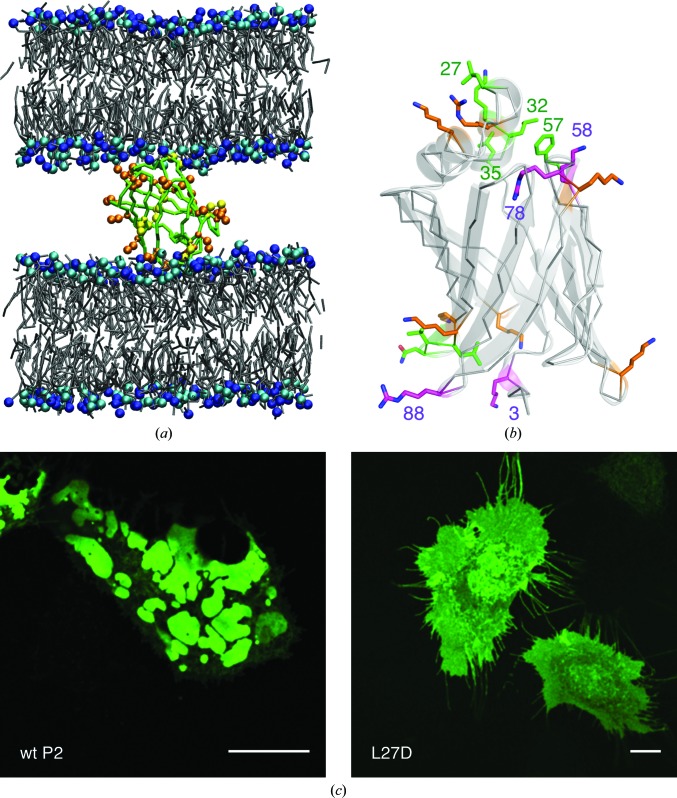
Membrane binding and stacking by P2 *in silico* and *in vivo*. (*a*) The end point (after 1.5 µs) of the CG MD simulation. The lower bilayer represents the pre-formed membrane, while the upper bilayer was formed during the simulation. (*b*) Mapping of contacts during the simulation. The most important polar contacts are indicated in magenta and the major nonpolar contacts are indicated in green. Further polar contacts are indicated in orange. Details are shown in Supplementary Fig. S1. (*c*) Formation of membrane domains by wild-type (left) and L27D mutant (right) P2 in cell culture. The scale bar is 10 µm in length.

To further elucidate the membrane-bound orientation of P2 and its interaction with two apposing lipid bilayers, coarse-grained MD simulations were carried out. As a starting configuration, P2 in a mixture of DPPC and DPPG was placed on top of a pre-equilibrated DPPC/DPPG (1:1) bilayer. During the formation of a double bilayer, P2 becomes sandwiched between the two membranes *via* its positively charged residues (Fig. 8 ▶ *a*), while the tip of the helical segment, including Leu27, inserts into the hydrophobic compartment of the bilayer. The stability of the simulation was judged by monitoring the distance between the centres of mass of the two bilayers, which stabilized within 800 ns of simulation time and was 75.24 ± 0.028 Å over the last 250 ns.

A number of residues are implicated in membrane binding through either electrostatic or hydrophobic interactions (Fig. 8 ▶ *b*, Supplementary Fig. S1). The two main hydrophobic residues involved are Leu27 (at the N-terminus of helix α2) and Phe57 (in the β3–β4 loop); additional hydrophobic contacts are made by residues in helix α2 (Leu32 and Leu35) and those in loop β8–β9 on the opposite face. A number of basic residues interact with the phospholipids during the simulation, with most contacts made by Lys3, Lys58, Arg78 and Arg88. A functional relevance in membrane binding can therefore be attributed to the Phe57–Lys58 dyad, which is likely to lie at the interface between the hydrophilic and hydrophobic layers of the membrane. The lack of Phe57 in FABP1 generates a more constantly open portal region, which is likely to be important for the diffusive transport mechanism. On the other hand, Arg78 is buried in the P2 crystal structure and salt-bridged to Asp76; it must swing open to fully contact phospholipids when interacting with the membrane surface. A comparative analysis of the FABP family (Fig. 5 ▶ *a*) indicates that whenever Arg78 is present Asp76 is also conserved, suggesting that this salt bridge, in contact with the bound fatty acid, is of functional importance. The residue making most hydrophilic contacts is Arg88, on the face opposite to the portal region. Interestingly, Arg88 is not conserved in other FABPs and could, together for example with Lys3, be important for the simultaneous binding of two apposing membranes, which is a unique feature of P2 in the FABP family.

### 3.7. Membrane-domain formation in a cellular environment   

MBP is able to induce membrane-sheet formation in oligodendrocytes and in cultured cells with heterologous expression systems (Aggarwal *et al.*, 2013 ▶). As P2, which is structurally unrelated to MBP, also has a high positive charge, binds to and stacks membranes *in vitro* and is localized in myelin, we employed a cell-culture model to assess whether P2 functions similarly to MBP in a cellular context. We used a previously established assay in which P2 is fused to the cytosolic domain of a transmembrane domain carrying GFP in its luminal part. Ptk2 cells were transfected with GFP–TM–P2 constructs and the formation of membrane domains was followed by fluorescence microscopy (Fig. 8 ▶ *c*). Similarly to MBP (Aggarwal *et al.*, 2013 ▶), wild-type P2 induced strong membrane-domain formation in this experimental system. The observed domains are generated between the membrane of the endoplasmic reticulum and the plasma membrane and are mediated by the cytosolic fusion partner. The L27D mutant, which directly affects the hydrophobic tip of the helical lid domain, failed to induce domain formation, but was instead localized evenly throughout the cell. This experiment further suggests a role for P2 in membrane binding and stacking, and confirms the importance of Leu27 and the hydrophobic tip for P2 function in an intracellular environment.

## 4. Concluding remarks   

We have provided a complete picture of the P2 fatty acid-binding cavity, which will also be instrumental in understanding ligand binding by other FABP family members. As an FABP, P2 is unique in being associated with the cytoplasmic leaflets of the PNS myelin membrane. We have highlighted the key determinants of membrane binding by human P2, including the insertion of Leu27 into the membrane, the anion-binding sites that are most likely to be involved in membrane binding and a number of key membrane-interacting residues, such as the Phe57–Lys58 dyad, as well as conformational changes upon membrane binding in the portal region.

A likely scenario that is compatible with the current data is as follows: P2 is attracted to the membrane *via* its surface potential and, when suitably positioned, the hydrophobic residues in the portal region, including Leu27 and Phe57, insert deeper into the membrane. Conformational changes follow, including exposure of Arg78 and partial unfolding of the helical lid. A trigger for such changes may lie in the anion-binding site next to the conserved Pro38, which may interact with membrane phospholipid headgroups. All these together will result in opening of the portal region, enabling ligand transfer. On the opposite side, through the second positively charged face including Arg88, P2 associates with another lipid bilayer. This arrangement stabilizes both the protein and the membrane and may be important for the long-term maintenance of myelin.

Many of these findings are also relevant when considering conformational changes in the portal region of other FABPs, especially those with a collisional transfer mechanism. P2 represents a highly accurate model system for studying protein–membrane interactions in general, as well as lipid bilayer stacking induced by myelin proteins in particular.

## Supplementary Material

PDB reference: human myelin peripheral membrane protein P2, 4bvm

Supplementary Figure 1. DOI: 10.1107/S1399004713027910/mh5108sup1.pdf

Supplementary Figure 2. DOI: 10.1107/S1399004713027910/mh5108sup2.pdf

## Figures and Tables

**Table 1:** Data-processing and structure-refinement statistics

Space group	*P*4_1_2_1_2
Unit-cell parameters (Å, °)	*a* = *b* = 58.07, *c* = 101.5, α = β = γ = 90
Wavelength (Å)	0.9444
Resolution range (Å)	20–0.93 (0.95–0.93)
〈*I*/σ(*I*)〉	22.5 (1.1)
*R*_merge_ (%)	3.6 (82.3)
*R*_meas_ (%)	3.9 (108.1)
Completeness (%)	98.2 (84.4)
Multiplicity	6.5 (1.8)
CC_1/2_ † (%)	100 (39.3)
Wilson *B* factor (Å^2^)	10.7
*R*_cryst_ (%)	10.4 (30.7)
*R*_free_ (%)	12.1 (33.3)
R.m.s.d., bond lengths (Å)	0.020
R.m.s.d., bond angles (°)	2.07
Average *B* factor (Å^2^)	
Protein	9.8
Ligand	10.9
Solvent	20.7
Ramachandran statistics, residues in‡ (%)	
Favoured region	98.5
Allowed region	100
*MolProbity* score‡	1.17 [91st percentile]
Mean anisotropy§	
Protein	0.43 ± 0.13
Ligand	0.43 ± 0.12
Solvent	0.37 ± 0.12

† CC_1/2_ is the correlation coefficient between two random half data sets (Karplus & Diederichs, 2012 ▶).

‡ As defined by *MolProbity* (Chen *et al.*, 2010 ▶).

§ As defined by *PARVATI* (Merritt, 1999 ▶).

## Footnotes

1 Supporting information has been deposited in the IUCr electronic archive (Reference: MH5108).
