# Supplementary material for: Atomic resolution view into the structure–function relationships of the human myelin peripheral membrane protein P2
Source: Acta Crystallogr D Biol Crystallogr. 2013 Dec 31;70(Pt 1):165–76. doi: 10.1107/S1399004713027910 (PMC3919267; doi:10.1107/S1399004713027910)
Supplement: Supplementary file 1 [file d-70-00165-sup1.pdf]

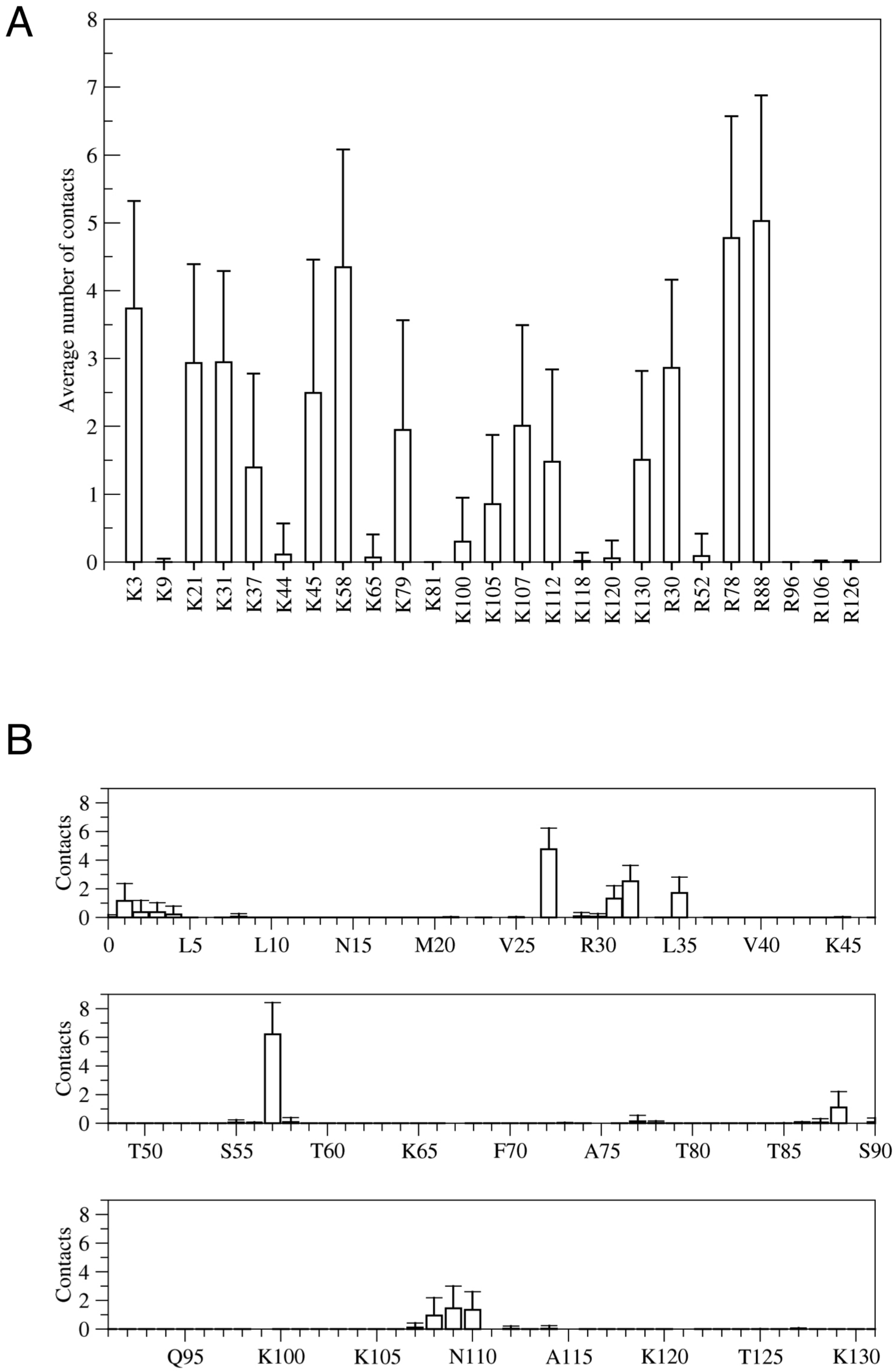

Supplementary Figure 1. Contacts between P2 and the lipid bilayers during the molecular dynamics simulation. A. Contacts between basic residues and phosphate groups in lipid headgroups during the simulation. B. Average number of contacts between the protein side chains and the coarse grained beads of the lipid tails. The contacts were calculated over the last 250 ns of the simulation. A contact is defined if a side-chain bead is within 6 Å of the bead of the lipid tail.
