# Supplementary material for: Atomic resolution view into the structure–function relationships of the human myelin peripheral membrane protein P2
Source: Acta Crystallogr D Biol Crystallogr. 2013 Dec 31;70(Pt 1):165–76. doi: 10.1107/S1399004713027910 (PMC3919267; doi:10.1107/S1399004713027910)
Supplement: Supplementary file 2 [file d-70-00165-sup2.pdf]

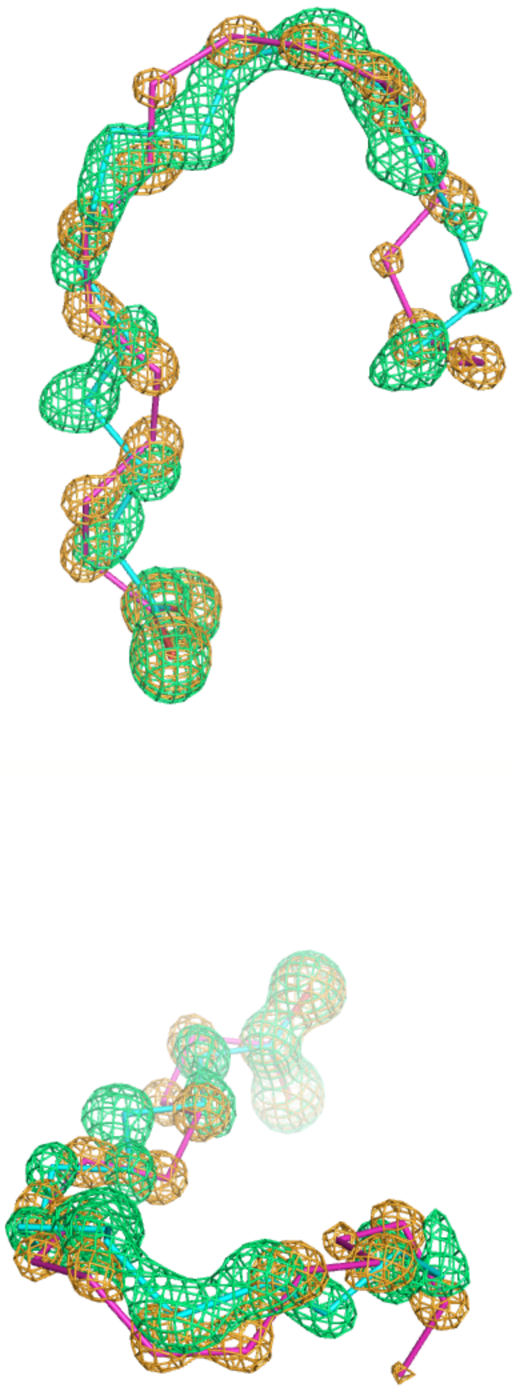

Supplementary Figure 2. Electron density maps calculated without the palmitate molecule. Palmitate is in cyan and *cis*-vaccenate in magenta. The maps are $2F_o$-F_c at 2.5 σ (orange) and F_o-F_c at 5.0 σ contour level. Top: side view, bottom: view turned approximately 100° about the x-axis to show the position of the double bond in *cis*-vaccenate.
